# Linear Variable Filter Hyperspectral Imaging for Determination of Acidity and Hardness of Multiple Fruits

**DOI:** 10.3390/foods15132348

**Published:** 2026-07-02

**Authors:** Yixuan Sun, Bo Li, Mei Sun, Yi Yang

**Affiliations:** School of Computer and Artificial Intelligence, Beijing Technology and Business University, Beijing 100048, China

**Keywords:** hyperspectral imaging, linear variable filter, fruit quality, pH, hardness, spectral index

## Abstract

Acidity determines the maturity of fruits and is an important component of fruit taste. Hardness is a key indicator for judging ripening status, storage tolerance, and transportation quality. Therefore, detecting acidity and hardness is of great significance. This article uses a linear gradient filter type hyperspectral imager to obtain hyperspectral data of apples, pears, and kiwifruit. A total of 150 fruits (50 apples, 50 pears, and 50 kiwifruits) were used. The dataset was split into modeling and validation sets in a 1:1 ratio using the concentration gradient (SPXY) method. Six spectral indices (NI, RI, DI, AI, TBNI, TBRI) were used to construct detection models for acidity and hardness across multiple fruit types, suitable for combined multi-fruit datasets. Results showed that the Three-Band Normalized Index (TBNI) achieved the highest accuracy for acidity prediction, with R^2^C = 0.832, R^2^V = 0.783, MAE = 0.14, and MRE = 0.03. The Three-Band Ratio Index (TBRI) achieved the highest accuracy for hardness prediction, with R^2^C = 0.914, R^2^V = 0.898, MAE = 0.63, and MRE = 0.19. These findings provide technical support for rapid detection of fruit physiological indicators in combined multi-fruit scenarios.

## 1. Introduction

Fruit maturity can be reflected by several physiological indicators, such as fruit firmness, soluble solids content, titratable acidity, and skin color [1,2,3,4,5,6]. In this study, acidity refers to pH-type acidity, which was measured using a calibrated pH meter. pH was chosen over titratable acidity (TA) because pH provides a rapid, non-destructive-compatible measurement that is directly correlated with fruit ripening and consumer perception of fruit taste. As fruit maturity increases, firmness decreases, soluble solid content increases, titratable acidity decreases, and skin color transitions from ground color to surface color. Hyperspectral imaging technology integrates traditional imaging techniques with spectroscopy. Its characteristic of combining spatial and spectral information enables the acquisition of surface feature information while detecting the internal quality of fruits. By calculating the parameter values of fruit maturity indicators at each pixel and employing pseudo-color image processing, visual prediction of fruit maturity can be achieved [7,8,9,10,11,12,13,14]. Liu Jinxiu et al. [15] used a near-infrared hyperspectral imaging system to investigate the hyperspectral information of small white apricots at four different maturity levels. By comparing the discrimination performance of full-waveband and feature-waveband spectra, different preprocessing methods, different sample set partitioning methods, and different modeling approaches, an optimal discriminant model for the maturity of small white apricots was established. The results showed that multiple optimal combinations exist for the qualitative discriminant model of small white apricot maturity, all achieving high recognition accuracy. Raj et al. [16] employed a hyperspectral imaging system to study strawberries at three maturity levels. A support vector machine (SVM) classification model for maturity was developed based on the full spectral range, achieving a classification accuracy exceeding 98%. Munera et al. [17] investigated the hyperspectral images of nectarines during ripening, and the results indicated that the coefficients of determination for both the ripening index (RPI) and the internal quality index (IQI) prediction models were greater than 0.87. Cao Xiaofeng et al. [18] studied the hyperspectral images of winter jujube at three maturity levels. By predicting the discriminant vectors of the Sis-PLS-DA model and visually displaying the results using different colors, visual grading of winter jujube maturity was achieved. However, current research on the detection of physiological indicators such as acidity and hardness in fruits using hyperspectral technology typically focuses on analyzing and modeling a single fruit type. Consequently, the resulting models are only suitable for detecting physiological indicators of a single fruit type and cannot be applied to the detection of physiological indicators in combined multi-fruit datasets involving multiple fruit types. The development of a unified model capable of simultaneously predicting acidity and hardness across different fruit types remains a significant challenge, as spectral characteristics vary substantially among fruit species due to differences in skin structure, pigment composition, and internal tissue properties. The linear variable filter (LVF) hyperspectral imager used in this study offers a promising alternative to conventional spectroscopic approaches, as it provides a cost-effective and portable solution for rapid spectral data acquisition without mechanical scanning components.

Currently, spectrometers used to study fruit maturity and physiological indicators are mainly classified into two types based on their spectroscopic methods: staring type and push-broom type [19]. Staring-type instruments use liquid crystal tunable filters or acousto-optic tunable filters for dispersion. They feature simple system design and are widely applicable, but suffer from drawbacks such as low and uneven spectral resolution, spectral range limitations due to material constraints, difficulty in achieving broad spectral coverage, spatial aberrations across different spectral bands, and an inability to image moving targets. Push-broom-type instruments typically use gratings or prisms for dispersion, simultaneously acquiring spatial and spectral information of samples. They offer high spectral resolution and uniform sampling. However, higher spectral resolution leads to reduced energy received by the system. Moreover, spectrometers using gratings or prisms have complex structures and high system costs, which limit their application in fruit quality detection. In contrast, spectral cameras based on linear variable filters utilize a special coating technology and do not require a separate dispersive spectrometer module, enabling continuous imaging of target objects across dozens or hundreds of spectral bands within the spectral coverage range.

This paper is based on linear variable filter-based hyperspectral imaging technology. By analyzing the spectra of different fruits (apple, pear, kiwifruit) and their corresponding physiological indicators such as acidity and hardness, and using the Normalized Index (NI), Ratio Index (RI), Difference Index (DI), Adjustable Index (AI), Three-Band Normalized Index (TBNI), and Three-Band Ratio Index (TBRI), we aim to construct unified detection models for acidity and hardness that are applicable across multiple fruit types, without requiring prior identification of fruit type. This is particularly relevant for practical sorting-line applications where rapid, non-destructive screening of mixed fruit batches is needed. This will provide technical support for the rapid detection of acidity and hardness of different fruits in combined multi-fruit datasets involving multiple fruit types.

## 2. Materials and Methods

### 2.1. Instruments and Equipment

**Figure 1:**
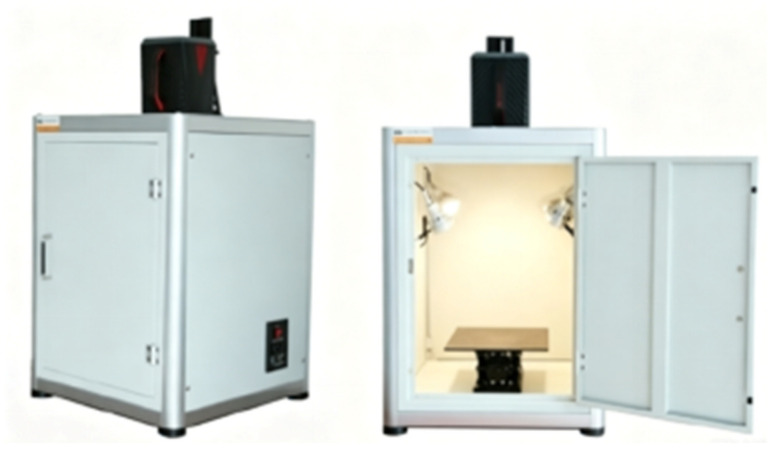
Hyperspectral imaging system.

Hyperspectral image data of different fruits were acquired using the Lambda-VN hyperspectral imager manufactured by Wuxi Spectrum Vision Technology Co., Ltd. (Wuxi, China). This imager is primarily composed of a linear variable filter, lens, camera, and other components. The fruit hyperspectral testing system mainly consists of the Lambda-VN hyperspectral imager, halogen light source, dark box, and computer, as shown in Figure 1. The hyperspectral images were acquired with an exposure time of 50 ms, object-camera distance of 30 cm, and illumination angle of 45 degrees. The spectral sampling interval was 2.2 nm, resulting in 256 spectral bands in the 420–1000 nm range. Prior to each acquisition session, the system was calibrated using a standard white reference board and a dark reference (obtained by covering the lens with a black cap). The halogen light source was preheated for 30 min before acquisition to ensure stable illumination. The parameter settings of the experimental instrument are listed in Table 1.

### 2.2. Experimental Materials

A total of 150 fruits were purchased from a local wholesale market in Beijing, China, including 50 apples (20 Fuji, 15 Gala, and 15 Golden Delicious), 50 pears (25 Ya Pear and 25 Crown Pear), and 50 kiwifruits (Hayward variety, originating from Shaanxi Province, China). All fruits were at commercial maturity stage, purchased on the same day, stored at 4 °C and 85% relative humidity, and measured within 48 h of purchase. It should be noted that refrigerated storage for 48 h may cause minor changes in fruit firmness and pH; however, these effects were expected to be minimal within this short storage period. The dataset was split into modeling and validation sets in a 1:1 ratio using the concentration gradient (SPXY) method, ensuring equal representation of each fruit type in both sets (25 fruits per type in each set). After washing and drying the 150 fruits, the Lambda-VN hyperspectral imager was used to acquire hyperspectral image data of each fruit. Meanwhile, a pH meter (PHS-3C, Leici, Shanghai, China) calibrated with pH 4.00, 6.86, and 9.18 standard buffers, and a fruit hardness tester (GY-4, Top Instrument, Hangzhou, China) with an 11.1 mm diameter probe, 10 mm penetration depth, and 1 mm/s penetration speed were used to measure the acidity (pH) and hardness (kgf) of the 150 fruits, respectively. All measurements were performed at room temperature (20 ± 2 °C). Three replicate measurements were taken per fruit at the equatorial region after peeling, and the pH and hardness readings were taken at the same equatorial region where the hyperspectral ROI was selected, ensuring spatial consistency between spectral and reference data.

### 2.3. Hyperspectral Image Preprocessing

The preprocessing of hyperspectral images mainly consisted of two parts: image correction and spectral calculation.

(1) Image correction: The acquired fruit hyperspectral images required black–white frame correction to obtain fruit reflectance data. The formula for black–white frame correction is as follows:

(1) $$R_{ref}=\frac{{DN}_{raw}-{DN}_{dark}}{DN_{white}-DN_{dark}}*R_{white}$$

where $R_{\mathrm{ref}}$ represents the reflectance value, ${\mathrm{DN}}_{\mathrm{raw}}$ represents the raw DN value of the data, $DN_{\mathrm{white}}$ represents the white reference DN value (obtained from a standard white calibration board), $DN_{\mathrm{dark}}$ represents the dark background noise of the instrument, and $R_{\mathrm{white}}$ represents the reflectance of the whiteboard. DN is an abbreviation for Digital Number, representing the pixel brightness value of the image, recording the grayscale value of the ground object. It is dimensionless and an integer value. Its magnitude is related to the sensor’s radiometric resolution, ground object emissivity, atmospheric transmittance, and scattering rate. R is an abbreviation for Reflectance, representing the percentage of radiant energy reflected by an object relative to the total incident radiant energy. Its value range is always less than or equal to 1. Reflectance can be used to determine the properties of objects.

(2) Spectral calculation: A region of interest (ROI) of 25 × 25 pixels was manually selected at the equatorial region of each fruit on the hyperspectral reflectance images. Three ROIs were selected per fruit to account for surface heterogeneity. For each fruit, the average spectrum was first computed within each individual ROI (by averaging all pixels within that ROI); the three ROI-level averages were then further averaged to obtain a single representative spectrum per fruit. Each fruit thus contributed one spectrum to the modeling dataset. The average spectrum of all pixels within the ROI was used as the reflectance spectrum of the fruit for modeling analysis with acidity and hardness.

### 2.4. Model Construction and Validation

The Normalized Index (NI), Ratio Index (RI), Difference Index (DI), Adjustable Index (AI), Three-Band Normalized Index (TBNI), and Three-Band Ratio Index (TBRI) were used to construct detection models for the acidity and hardness of multiple fruits based on linear variable filter hyperspectral technology. Using acidity and hardness as the reference, the modeling set and validation set were selected in a 1:1 ratio using the concentration gradient method (as shown in Table 2). Fruit-type stratification was ensured, with equal numbers of each fruit type in both sets. Additionally, 5-fold cross-validation was performed to assess model robustness, and performance metrics were reported separately for apple, pear, kiwifruit, and the combined dataset. Accuracy evaluation was performed based on the coefficient of determination (R^2^), mean absolute error (MAE), and mean relative error (MRE) (where a larger R^2^ and smaller MAE and MRE indicate higher model accuracy), and the best methods for detecting acidity and hardness of multiple fruits were selected.

The calculation formulas for the Normalized Index (NI), Ratio Index (RI), Difference Index (DI), Adjustable Index (AI), Three-Band Normalized Index (TBNI), and Three-Band Ratio Index (TBRI) are shown in Equations (1)–(6):

(2) $$NI=(R_{i}-R_{j})/(R_{i}+R_{j})$$

(3) $$RI=R_{i}/R_{j}$$

(4) $$DI=R_{i}-R_{j}$$

(5) $$AI=1.5*(R_{i}-R_{j})/(R_{i}+R_{j}+0.5)$$

(6) $$TBNI=(R_{i}-R_{j}-R_{k})/(R_{i}+R_{j}-R_{k})$$

(7) $$TBRI=R_{i}/(R_{j}+R_{k})$$

where i, j, and k represent arbitrary wavelength positions within the 420–1000 nm range, and $R_{i}$, $R_{j}$ and $R_{k}$ represent the spectral reflectances at the corresponding wavelengths.

The calculation formulas for the coefficient of determination (R^2^), mean absolute error (MAE), and mean relative error (MRE) are as follows:

(8) $$R^{2}=1-\frac{\sum_{i=1}^{n}({\hat{y}}_{i}-y_{i}{)}^{2}}{\sum_{i=1}^{n}({\bar{y}}_{i}-y_{i}{)}^{2}}$$

(9) $$MAE=\frac{1}{N}\sum_{i=1}^{N}|y_{i}-{\hat{y}}_{i}|$$

(10) $$MRE=\frac{1}{N}\sum_{i=1}^{N}\left|\frac{y_{i}-{\hat{y}}_{i}}{y_{i}}\right|$$

where N is the number of samples, $y_{i}$ is the measured value of the i-th sample, ${\hat{y}}_{i}$ is the predicted value of the i-th sample, and $\bar{y}$ is the mean of the measured values of all samples.

## 3. Results and Analysis

### 3.1. Spectral Reflectance Changes in Different Fruits

**Figure 2:**
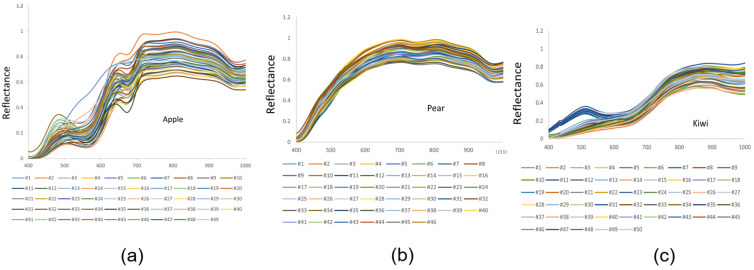
Spectral reflectance curve (**a**): apple; (**b**): pear; (**c**): kiwifruit.

The spectral reflectances of fruits (apple, pear, and kiwifruit) were obtained after steps including black–white frame correction and spectral calculation of the hyperspectral images, as shown in Figure 2. From Figure 2, it can be seen that the spectral reflectance of apples within the 420–1000 nm range exhibits relatively distinct “peak” and “valley” positions. One “peak” is located near 500 nm, and two “valleys” are located near 560 nm and 680 nm, respectively. The peak near 500 nm is associated with green reflectance, as chlorophyll absorption is relatively weak in this region. The valley near 560 nm corresponds to carotenoid absorption, while the valley near 680 nm is attributed to strong chlorophyll a absorption in the red region. These absorption features are directly related to fruit ripening, as chlorophyll degrades, carotenoid content changes, and organic acid metabolism shifts during maturation, all of which modify the spectral reflectance pattern. The spectral reflectance of pears within the 420–1000 nm range shows no obvious “peak” or “valley” positions, but within the 420–620 nm range, the spectral reflectance exhibits a characteristic of strong reflectance steep change, which is related to the combined effects of pigment absorption and scattering in the fruit skin. For kiwifruits within the 420–1000 nm range, some kiwifruits show an obvious “peak” near 500 nm while others do not. This is because green-skinned kiwifruits, due to weak green absorption, form a “green peak”.

### 3.2. Correlation Analysis Between Mixed Fruits and Single-Band Reflectance

**Figure 3:**
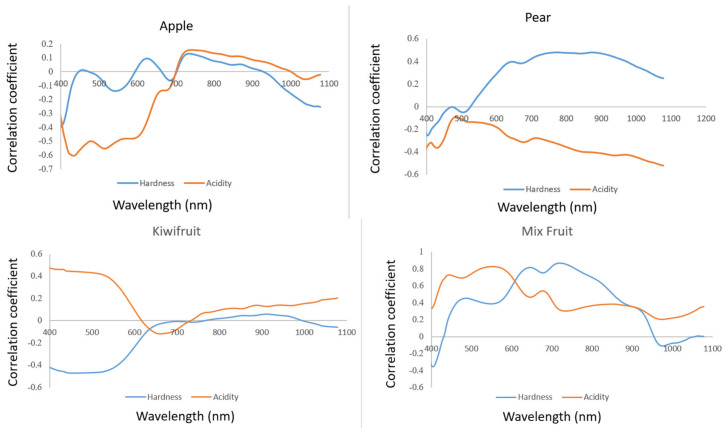
Correlation coefficient between hyperspectral reflectance and hardness, acidity.

Figure 3 shows the correlation coefficients between the spectral reflectance of mixed fruits and hardness and acidity within the 420–1000 nm range. The correlation coefficient was calculated using the Pearson correlation coefficient (PCC), which measures the degree of correlation between two variables X and Y, with values ranging between −1 and 1. The PCC was calculated as r = cov(X,Y)/(σ_X × σ_Y), where cov(X,Y) is the covariance between variables X and Y, and σ_X and σ_Y are their standard deviations. Correlation strengths were evaluated based on the absolute r values, where |r| ≥ 0.7 indicates strong correlation, 0.5 ≤ |r| < 0.7 indicates moderate correlation, and |r| < 0.5 indicates weak correlation. Separate correlation analyses were performed for each fruit type.

Figure 3 shows the Pearson correlation coefficients (r) between hyperspectral reflectance and hardness and acidity for apples, pears, kiwifruits, and their mixed samples in the 420–1000 nm range. Hyperspectral reflectance, hardness and acidity are shown as a function of wavelength.

As shown in the figure, the absolute correlation coefficients for apple hardness are below 0.4 across the entire spectral range, indicating weak overall linear correlation. Near 420 nm, a moderate negative correlation is observed; the correlation increases rapidly with wavelength and shifts from negative to positive around 450 nm. In the 600–700 nm long-wave visible region, a positive correlation peak is reached. After 700 nm, the correlation decreases continuously, and turns negative again after 900 nm. For apple acidity, significant differences are observed across the entire spectral range. In the 420–650 nm visible region, a moderately strong negative correlation is observed, with the negative correlation peak at 420 nm. This is because this region corresponds to the strong absorption bands of chlorophyll and carotenoids, and acidity changes are highly coordinated with pigment degradation and organic acid metabolism during fruit ripening. After 650 nm, the correlation coefficient increases rapidly, shifting from negative to positive near 720 nm, and reaching a weak positive correlation peak at 750 nm. In the long-wave near-infrared region, the correlation gradually decreases, approaching zero at 1000 nm.

Pear is the sample with the strongest hardness correlation among the three single varieties. In the 420–550 nm range, the correlation is weak and fluctuates near zero. After 550 nm, the correlation coefficient increases rapidly, maintaining a moderately strong positive correlation in the 600–900 nm range, with a peak in the 800–850 nm near-infrared region. After 900 nm, the correlation decreases slightly, but remains moderately positive at 1000 nm. This pattern is highly consistent with the near-infrared spectral response to cell wall pectin degradation and cellular structural changes during pear ripening. Pear acidity shows a negative correlation across the entire spectral range, with the correlation strength gradually increasing with wavelength. At 420 nm, a moderate negative correlation is observed. After 500 nm, the correlation decreases continuously, stabilizing at a moderate negative correlation level in the 700–1000 nm near-infrared region, with the negative correlation peak at 1000 nm.

Kiwifruit hardness shows significant negative correlation in the short-wave region and very weak correlation in the long-wave region. In the 420–500 nm range, a moderately strong negative correlation is observed, reaching a minimum at 450 nm. After 500 nm, the correlation increases continuously, shifting from negative to positive near 680 nm. In the 700–1000 nm near-infrared region, only a very weak positive correlation is maintained. The correlation trend for kiwifruit hardness is completely opposite to that of apples and pears, making it the only fruit among the three single varieties that shows a positive correlation in the visible region. At 420 nm, a strong positive correlation is observed. From 420 to 600 nm, the correlation decreases continuously, approaching zero at 600 nm. Near 650 nm, a weak negative correlation minimum appears. After 700 nm, the correlation gradually increases, returning to a weak positive correlation at 1000 nm. This difference is directly related to the optical properties of kiwifruit flesh with high chlorophyll content and the organic acid metabolism pattern dominated by citric acid.

The mixed samples of apples, pears, and kiwifruits show significantly higher correlation strengths between hardness, acidity, and spectral reflectance than any single variety, with positive correlation dominating across the entire spectral range. For hardness, the correlation is near zero at 420 nm, then increases rapidly. The first positive correlation peak is reached at 480 nm. After 650 nm, the correlation increases rapidly again, reaching the highest correlation coefficient in the entire figure at 720 nm. In the 700–850 nm range, the correlation coefficient remains above 0.8. After 900 nm, the correlation decreases rapidly, shifting from positive to negative near 920 nm. For acidity, positive correlation is observed across the entire spectral range. At 420 nm, a strong positive correlation is already reached. The peak is reached in the 520 nm visible region. After 550 nm, the correlation gradually decreases, with a local minimum at 700 nm. After 750 nm, the correlation increases to approximately 0.35, forming a plateau. After 900 nm, the correlation decreases slowly again, maintaining a weak positive correlation at 1000 nm.

### 3.3. Analysis of Acidity Models for Mixed Fruits

Using the spectral reflectances of 150 mixed fruits (apples, pears, kiwifruits), the Normalized Index (NI), Ratio Index (RI), Difference Index (DI), Adjustable Index (AI), Three-Band Normalized Index (TBNI), and Three-Band Ratio Index (TBRI) were constructed respectively. MATLAB R2021b (MathWorks, USA) was used to select the optimal wavelength combinations for predicting acidity and hardness. A brute-force search was performed across all possible two-band and three-band combinations within the 420–1000 nm range at 10 nm intervals. For each combination, the R^2^ value was calculated using the modeling set only. The combination yielding the highest R^2^ was selected as optimal. To prevent overfitting, several measures were implemented: (1) wavelength selection was performed exclusively on the modeling set, and the validation set was used only for final model evaluation; (2) the search interval was set to 10 nm rather than the full spectral resolution (2.2 nm), reducing the number of candidate combinations and the risk of chance correlations; (3) a simple univariate linear regression model was adopted, which has lower overfitting risk compared to multivariate or nonlinear models; and (4) 5-fold cross-validation was performed on the modeling set to assess model stability.

**Figure 4:**
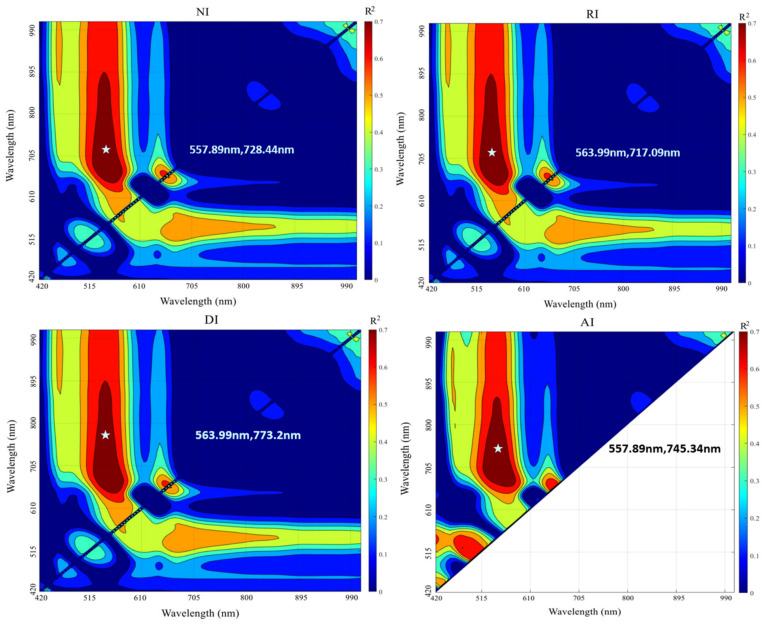
Spectral indices (NI, RI, DI, and AI) and R2 value of fruit acidity.

Taking the two-band spectral indices NI, RI, DI, and AI as examples, Figure 4 shows the R^2^ value distribution maps of the spectral index combinations (NI, RI, DI, and AI) constructed from mixed fruits within the 420–1000 nm spectral range against mixed fruit acidity. The color scale transitions from dark blue to dark red. Bluer positions of the spectral index wavelength combinations indicate lower R^2^ values and poorer fitting effects, while redder positions indicate higher R^2^ values and better fitting effects.

According to the screening results, the sensitive regions for predicting acidity using the spectral indices NI, RI, DI, and AI constructed from mixed fruits are similar. The optimal wavelength combinations for predicting fruit acidity were: 557.89 nm and 728.44 nm for NI; 563.99 nm and 717.09 nm for RI; 563.99 nm and 773.2 nm for DI; and 557.89 nm and 745.34 nm for AI.

Following the same method as the two-band indices NI, RI, DI, and AI, the optimal wavelength combinations for the three-band indices TBNI and TBRI for predicting acidity were screened. The optimal wavelength combination for the Three-Band Normalized Index (TBNI) for predicting fruit acidity was 563.99 nm, 793.72 nm, and 811.56 nm. The optimal wavelength combination for the Three-Band Ratio Index (TBRI) for predicting fruit acidity was 577.89 nm, 750.95 nm, and 811.56 nm.

Table 3 summarizes the calibration and validation results of acidity prediction models for apples, pears, kiwifruits, and mixed fruits based on six spectral indices (NI, RI, DI, AI, TBNI, TBRI)with optimal wavelength combinations. The evaluation metrics include the coefficient of determination for the calibration set (R^2^C), the coefficient of determination for the validation set (R^2^V), mean absolute error (MAE), and mean relative error (MRE).

From the validation set coefficient of determinationR^2^V, the optimal modeling methods vary significantly among different fruits: the mixed fruit sample shows the best prediction performance, with the TBNI model achieving the highest accuracy (R^2^V = 0.783, MAE = 0.14, MRE = 0.03), and the NI, RI, and AI models also showed excellent performance (R^2^V > 0.76), indicating that the mixed sample breaks the spectral variability limitations of single varieties and enhances the statistical robustness of the regression model. Apple ranks second, with the optimal model being the TBNI (R^2^V = 0.471, MAE = 0.423), but the overall accuracy is lower than that of the mixed sample, indicating that the spectral response of apple acidity has strong cultivar-specific characteristics that simple linear combinations cannot fully capture. The prediction accuracy for pear and kiwifruit is generally low, with all models showing R^2^V not exceeding 0.30 (the optimal model for pear is TBNI, R^2^V = 0.275; the optimal models for kiwifruit are DI/AI, R^2^V≈ 0.24), with relatively large MAE and MRE values, indicating that the acidity information of these two fruits may be coupled with strong absorption signals such as water and pigments within the current hyperspectral range, making it difficult for single difference or ratio indices to effectively extract features.

For both apple and mixed fruit samples, the TBNI achieved the highest R^2^V (apple: 0.471, mixed: 0.783), which is significantly higher than the corresponding NI/RI/DI/AI. This is attributed to the three-band combination (e.g., apple: 451.9, 439.1, 551.8 nm; mixed: 563.99, 793.72, 811.56 nm), which can effectively eliminate baseline drift and particle scattering interference through differential-ratio operations, thereby amplifying weak absorption features related to organic acids (such as malic acid and citric acid).

**Figure 5:**
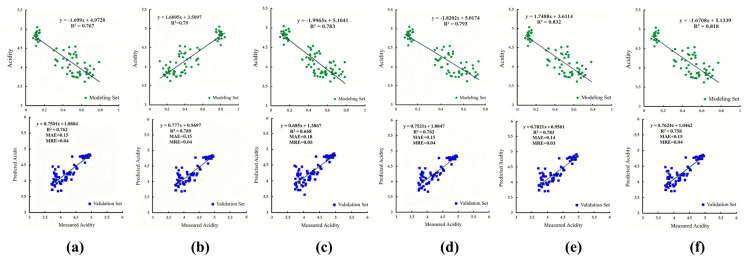
Fit curve between different indices and fruit acidity, and the 1:1 plot of predicted vs. actual values: (**a**) Normalized Index (NI); (**b**) Ratio Index (RI); (**c**) Difference Index (DI); (**d**) Adjustable Index (AI); (**e**) Three-Band Normalized Index (TBNI); (**f**) Three-Band Ratio Index (TBRI).

Figure 5 shows the fitting curves and 1:1 plots of predicted versus actual acidity values for the six optimal spectral index models.

### 3.4. Analysis of Hardness Models for Mixed Fruits

Similar to the method used for screening optimal spectral index combinations for acidity detection in mixed fruits, six spectral indices—including four dual-band indices (NI, RI, DI, AI) and two three-band indices (TBNI, TBRI)—were employed to identify the best combinations for predicting fruit firmness. The Normalized Index (NI) achieved optimal fruit firmness prediction using the wavelength combination of 750.95 nm and 795.21 nm; the ratio index (RI) using 756.53 nm and 784.24 nm; the difference index (DI) using 762.1 nm and 778.73 nm; the adjustable index (AI) using 756.53 nm and 784.24 nm; the Three-Band Normalized Index (TBNI) using 750.95 nm, 789.93 nm, and 916.96 nm; and the Three-Band Ratio Index (TBRI) using 406.92 nm, 728.44 nm, and 854.46 nm.

Table 4 summarizes the calibration and validation results for firmness prediction of apples, pears, kiwifruits, and mixed fruits using the optimal wavelength combinations based on the six spectral indices (NI, RI, DI, AI, TBNI, TBRI).

Compared with the previous acidity prediction results (Table 3), the accuracy of firmness prediction models for all fruits was significantly improved. Particularly, the validation set coefficient of determination (R^2^V) for kiwifruits and pears reached 0.58–0.99, while those for apples and mixed fruits generally exceeded 0.26–0.89. This indicates that hyperspectral reflectance is far more sensitive to fruit firmness (a textural property) than to acidity (a chemical property), likely because firmness is directly related to cell structure, water distribution, and physical scattering characteristics of cell wall polysaccharides, resulting in more pronounced spectral responses.

For apple firmness, R^2^V values of all models ranged from 0.268 to 0.354, with the TBNI being the optimal index (R^2^V = 0.354, MAE = 0.009, MRE = 0.754) using characteristic wavelengths of 992.0, 682.7, and 996.8 nm. This combination covers the near-infrared water absorption peak (~970 nm) and the red edge region (~680 nm), suggesting that apple firmness is primarily influenced by water status and chlorophyll degradation products. Although the DI model exhibited high calibration accuracy (R^2^C = 0.720), its validation accuracy decreased (R^2^V = 0.336), indicating slight overfitting.

Pear firmness prediction models performed excellently, with the AI (R^2^V = 0.702, MAE = 0.010, MRE = 0.314) and TBNI (R^2^V = 0.714, MAE = 0.010, MRE = 0.330) being optimal. The wavelength combinations concentrated in the blue-violet region (426–502 nm) and green region (521–576 nm), which are associated with UV–visible absorption by carotenoids, anthocyanins, and cell wall pectin, implying that pear firmness changes are accompanied by epidermal pigment degradation processes. Notably, DI performed poorly (R^2^V = 0.409) when using 992/996 nm (near-infrared), indicating that pear firmness information is more abundant in the visible region rather than the water absorption region.

All prediction models for kiwifruit firmness exhibited exceptionally high accuracy. The TBNI achieved nearly perfect validation performance (R^2^V = 0.987, MAE = 0.003, MRE = 0.367), with R^2^V even slightly higher than R^2^C (0.980), demonstrating strong generalization capability. The characteristic wavelengths were concentrated in the green-yellow region (527–552 nm) and blue-violet region (432–439 nm), indicating that kiwifruit firmness is highly correlated with chlorophyll decomposition and intercellular space changes. The RI model (R^2^V = 0.953) and TBRI (R^2^V = 0.954) also performed excellently, suggesting that ratio-based indices can effectively eliminate scattering noise.

Mixed fruit firmness prediction models demonstrated good accuracy, with all models achieving R^2^V values between 0.886 and 0.898. The TBNI (0.898) and TBRI (0.898) were optimal, with MAE ranging from 0.63 to 0.66 and MRE from 0.19 to 0.23. The wavelength combinations were concentrated in the near-infrared region (750–917 nm) and shortwave near-infrared, which are sensitive to water and structural scattering. Unlike acidity prediction, the firmness models for mixed fruits were not significantly superior to those for single varieties (e.g., kiwifruit), but overall maintained high predictability.

### 3.5. Stability Verification of Fruit Acidity and Hardness Detection Algorithm

As shown in Table 3, the Three-Band Normalized Index (TBNI) achieved the highest accuracy for predicting acidity in mixed fruits. To further verify the stability and robustness of the TBNI algorithm in acidity detection for mixed fruits and avoid overfitting issues, this study employed the commonly used evaluation technique of “five-fold cross-validation” to validate the performance and generalization capability of the TBNI algorithm. The main steps are as follows: (1). Dataset partitioning: A total of 150 samples including apples, pears, and kiwifruits were randomly and evenly divided into five subsets; (2). Model training and testing: Each subset was sequentially selected as the test set (30 samples), while the remaining subsets served as the training set (120 samples) for model training. This process was repeated 5 times to ensure each subset was used as the test set exactly once; (3). Performance evaluation: The accuracy of the five tests was recorded and averaged to obtain the model performance evaluation results. The cross-validation results are shown in Table 5.

As can be seen from Table 5, the R^2^C values of the training sets across the five tests ranged from 0.881 to 0.912, with a mean value of 0.893; the R^2^V values of the validation sets ranged from 0.788 to 0.924, with a mean value of 0.867; the MAE values of the validation sets ranged from 0.538 to 0.768, with a mean value of 0.656; and the MRE values of the validation sets ranged from 0.185 to 0.222, with a mean value of 0.197. These results fully demonstrate that the TBNI exhibits high accuracy, stability, and robustness in predicting acidity for mixed fruits.

Similarly, as shown in Table 4, the Three-Band Ratio Index (TBRI) achieved the highest accuracy for predicting firmness in mixed fruits. To further verify the stability and robustness of the TBRI algorithm in firmness detection for mixed fruits and avoid overfitting issues, this study employed the commonly used evaluation technique of “five-fold cross-validation” to validate the performance and generalization capability of the TBRI algorithm. The specific procedure was consistent with that used for validating the TBNI algorithm, and the cross-validation results are shown in Table 6. As can be seen from Table 6, the R^2^C values of the training sets across the five tests ranged from 0.767 to 0.824, with a mean value of 0.791; the R^2^V values of the validation sets ranged from 0.600 to 0.859, with a mean value of 0.768; the MAE values of the validation sets ranged from 0.007 to 0.011, with a mean value of 0.008; and the MRE values of the validation sets ranged from 0.417 to 0.903, with a mean value of 0.697. These results fully demonstrate that the TBRI exhibits high accuracy, stability, and robustness in predicting firmness for mixed fruits.

## 4. Conclusions

This study used a linear variable filter-based hyperspectral imager to obtain spectral reflectances of different fruits (apples, pears, kiwifruits). Spectral index methods including NI, RI, DI, AI, TBNI, and TBRI were used to select optimal wavelength combinations for predicting the acidity and hardness of different fruits, and independent data were used for validation. The following conclusions were drawn:

(1) Within the 420–1000 nm range, the spectral reflectance of apples exhibits relatively distinct “peak” and “valley” positions; the spectral reflectance of pears shows no obvious “peak” or “valley” positions, but exhibits a characteristic of strong reflectance steep change within the 420–620 nm range; the skin of kiwifruits is green, and due to weak green absorption, their spectral reflectance shows an obvious “peak” near 500 nm.

(2) Within the 420–1000 nm range, the correlation coefficients between the spectral reflectance of mixed fruits and acidity are positively correlated, with the highest correlation concentrated in the 450–570 nm range. The correlation with hardness is positively correlated between 430 nm and 950 nm, with the highest correlation concentrated in the 650–750 nm range.

(3) Among the six spectral indices, the TBNI achieved the highest accuracy for predicting fruit acidity, with R^2^C of 0.832, R^2^V of 0.783, MAE of 0.14, and MRE of 0.03. The expression for predicting fruit acidity was y = 1.7488x + 3.6114 (where y is fruit acidity and x is the TBNI).

(4) Among the six spectral indices, the TBRI achieved the highest accuracy for predicting fruit hardness, with R^2^C of 0.914, R^2^V of 0.898, MAE of 0.63, and MRE of 0.19. The expression for predicting fruit hardness was y = 12.628x − 5.9445 (where y is fruit hardness and x is the TBRI).

The research results demonstrate that linear variable filter-based hyperspectral technology can achieve the prediction of acidity and hardness of different fruits, thereby providing technical support for the rapid detection of acidity and hardness of different fruits in combined multi-fruit datasets such as multiple fruit types. However, this study has several limitations. The sample size of 150 fruits (50 per species) is relatively small, and external validation using independent batches from different growing seasons or regions was not performed. Only pH-type acidity was measured; titratable acidity was not investigated. Additionally, the spectral index-based linear approach may not fully capture nonlinear relationships. Future work should include larger and more diverse sample sets, incorporate external validation, explore machine learning methods (e.g., PLSR, SVR) for comparison, and extend the framework to a broader range of fruit species.

## Figures and Tables

**Table 1:** Parameters of Lambda-VN camera of linear gradient filter hyperspectral imager.

Parameter Category	Parameter Value	Parameter Category	Parameter Value
Spectral Range	420–1000 nm	Spectral Resolution	10 nm
Number of Spectral Channels	>100	Focal Length	25 mm/35 mm/50 mm
Detector	2048 × 2048 (CMOS)	Pixel Count (Spatial Dimension × Scanning Dimension)	1600 × 1200
Pixel Size	5.5 × 5.5 μm	Frame Rate	90 fps
Digital Output	10 bit	Exposure Time Range	28 μs–1 s

**Table 2:** Statistical analysis of data for fruit acidity and hardness modeling and validation sets.

Biochemical Indicator	Dataset	Number	Maximum	Minimum	Mean	Standard Deviation
Acidity (pH)	Total	150	5.042	3.621	4.280	0.418
	Modeling Set	75	5.042	3.621	4.280	0.422
	Validation Set	75	4.967	3.702	4.280	0.415
Hardness (kgf)	Total	150	9.21	0.89	4.868	2.532
	Modeling Set	75	9.21	0.89	4.865	2.552
	Validation Set	75	8.77	0.98	4.870	2.513

**Table 3:** Evaluation of accuracy in predicting fruit acidity using different modeling methods.

Types of Fruits	Model Method	Wavelength Combination (nm)	R^2^C	R^2^V	MAE	MRE	Regression Equation (y Stands for Predicted Acidity)
Apple	NI	773.2, 778.7	0.211	0.108	0.543	0.102	y = 496.628 ∗ NI + 7.785
	RI	773.2, 778.7	0.208	0.114	0.528	0.106	y = 249.156 ∗ RI – 241.358
	DI	751.0, 843.8	0.177	0.087	0.557	0.103	y = 21.815 ∗ DI + 6.961
	AI	773.2, 778.7	0.245	0.214	0.507	0.0971	y = 452.339 ∗ AI + 7.813
	TBNI	451.9, 439.1, 551.8	0.675	0.471	0.423	0.074	y = 0.061 ∗ TBNI + 6.779
	TBRI	875.5, 880.8, 870.3	0.316	0.189	0.515	0.098	y = 3753.827 ∗ TBRI – 1869.655
Pear	NI	624.1, 630.0	0.226	0.148	0.837	0.132	y = −565.49 ∗ NI + 3.721
	RI	630.0, 600.2	0.228	0.169	0.803	0.126	y = 45.333 ∗ RI – 41.546
	DI	576.1, 641.8	0.286	0.151	0.737	0.114	y = −32.234 ∗ DI + 2.691
	AI	570.1, 630.0	0.252	0.204	0.734	0.114	y = −45.578 ∗ AI + 2.505
	TBNI	496.1, 477.2, 932.2	0.494	0.275	0.716	0.114	y = 0.0068 ∗ TBNI + 6.197
	TBRI	521.0, 618.2, 483.5	0.284	0.092	0.688	0.107	y = −51.702 ∗ TBRI + 30.764
Kiwi Fruit	NI	636.0, 647.7	0.270	0.093	0.526	0.214	y = −49.54 ∗ NI + 0.048
	RI	647.7, 636.0	0.271	0.093	0.514	0.225	y = 23.433 ∗ RI – 23.348
	DI	838.5, 843.8	0.235	0.245	0.554	0.201	y = −154.467 ∗ DI + 0.813
	AI	838.5, 843.8	0.223	0.242	0.516	0.224	y = −164.776 ∗ AI + 0.912
	TBNI	451.9, 947.4, 891.2	0.446	0.145	0.491	0.183	y = −0.001 ∗ TBNI + 1.368
	TBRI	653.6, 659.4, 641.8	0.327	0.128	0.517	0201	y = 229.535 ∗ TBRI – 114.658
Fruit Mix	NI	557.89, 728.44	0.767	0.762	0.15	0.04	y = −1.699 ∗ NI + 4.9728
	RI	563.99, 717.09	0.790	0.789	0.15	0.04	y = 1.6095 ∗ RI + 3.5097
	DI	563.99, 773.20	0.783	0.668	0.18	0.05	y = −1.9965 ∗ DI + 5.1041
	AI	557.89, 745.34	0.793	0.762	0.15	0.04	y = −1.8202 ∗ AI + 5.0174
	TBNI	563.99, 793.72, 811.56	0.832	0.783	0.14	0.03	y = 1.7488 ∗ TBNI + 3.6114
	TBRI	577.89, 750.95, 811.56	0.818	0.758	0.15	0.04	y = −1.6708 ∗ TBRI + 5.1339

**Table 4:** Evaluation of accuracy in predicting fruit Hardness using different modeling methods.

Types of Fruits	Model Method	Wavelength Combination (nm)	R^2^C	R^2^V	MAE	MRE	Regression Equation (y Stands for Predicted Acidity)
Apple	NI	992.0, 996.8	0.660	0.268	0.013	0.780	y = −8.062 ∗ NI − 0.003
	RI	996.8, 992.0	0.661	0.269	0.012	0.779	y = 4.017 ∗ RI − 4.02
	DI	992.0, 996.8	0.720	0.336	0.011	0.811	y = −6.147 ∗ D − 0.004
	AI	992.0, 996.8	0.679	0.289	0.012	0.797	y = −7.459 ∗ AI − 0.003
	TBNI	992.0, 682.7, 996.8	0.724	0.354	0.009	0.754	y = −1.637 ∗ TBNI − 1.639
	TBRI	982.2, 996.8, 977.2	0.672	0.278	0.012	0.871	y = −8.32 ∗ TBRI + 4.154
Pear	NI	426.3, 470.9	0.707	0.587	0.012	0.435	y = 0.189 ∗ NI + 0.137
	RI	426.3, 470.9	0.721	0.624	0.011	0.392	y = 0.233 ∗ RI − 0.035
	DI	992.0, 996.8	0.440	0.409	0.012	0.335	y = −10.919 ∗ DI − 0.009
	AI	426.3, 502.3	0.852	0.702	0.010	0.314	y = 0.539 ∗ AI + 0.314
	TBNI	521.0, 576.1, 426.3	0.849	0.714	0.010	0.330	y = −0.699 ∗ TBNI − 0.119
	TBRI	521.0, 545.7, 426.3	0.823	0.664	0.010	0.366	y = −0.696 ∗ TBRI + 0.56
Kiwi Fruit	NI	426.3, 527.2	0.903	0.931	0.005	1.095	y = 0.155 ∗ NI + 0.12
	RI	426.3, 521.0	0.931	0.953	0.004	0.920	y = 0.199 ∗ RI − 0.026
	DI	992.0, 996.8	0.903	0.934	0.005	0.952	y = −17.877 ∗ DI − 0.013
	AI	992.0, 996.8	0.871	0.920	0.006	1.033	y = −24.633 ∗ AI − 0.016
	TBNI	527.2, 545.7, 432.7	0.980	0.987	0.003	0.367	y = −0.349 ∗ TBNI − 0.051
	TBRI	533.3, 551.8, 439.1	0.967	0.954	0.005	0.770	y = −0.721 ∗ TBRI + 0.552
Fruit Mix	NI	750.95, 795.21	0.900	0.888	0.67	0.23	y = −66.934 ∗ NI + 7.2981
	RI	756.53, 784.24	0.904	0.892	0.66	0.22	y = 28.985 ∗ RI − 21.724
	DI	762.1, 778.73	0.916	0.886	0.65	0.21	y = −151.65 ∗ DI + 7.7403
	AI	756.53, 784.24	0.907	0.894	0.65	0.22	y = −99.028 ∗ AI + 7.4161
	TBNI	750.95, 789.93, 916.96	0.917	0.898	0.66	0.23	y = 26.15 ∗ TBNI − 18.523
	TBRI	406.92, 728.44, 854.46	0.914	0.898	0.63	0.19	y = 12.628 ∗ TBRI − 5.9445

**Table 5:** Five-fold cross-validation results of fruit acidity.

Number of Folds	Wavelength Combination (nm)	R^2^C	R^2^V	MAE	MRE
Fold 1	972.3, 671.1, 728.4	0.890	0.888	0.601	0.185
Fold 2	972.3, 676.9, 728.4	0.912	0.788	0.745	0.197
Fold 3	778.7, 911.8, 751.0	0.882	0.914	0.631	0.190
Fold 4	800.7, 932.2, 751.0	0.900	0.821	0.768	0.192
Fold 5	784.2, 911.8, 751.0	0.881	0.924	0.538	0.222
Average	-	0.893	0.867	0.656	0.197

**Table 6:** Five-fold cross-validation results of fruit hardness.

Number of Folds	Wavelength Combination (nm)	R^2^C	R^2^V	MAE	MRE
Fold 1	426.3, 854.5, 564.0	0.803	0.741	0.009	0.747
Fold 2	426.3, 859.8, 557.9	0.767	0.859	0.008	0.657
Fold 3	426.3, 859.8, 551.8	0.774	0.843	0.007	0.761
Fold 4	426.3, 854.5, 527.2	0.824	0.600	0.011	0.903
Fold 5	426.3, 854.5, 557.9	0.787	0.798	0.007	0.417
Average	-	0.791	0.768	0.008	0.697

## Data Availability

The original contributions presented in this study are included in the article. Further inquiries can be directed to the corresponding author.
